# Anomalies de fermeture du tube neural en République centrafricaine : mortalité élevée et opportunités manquées pour l’ODD 3

**DOI:** 10.48327/mtsi.v6i3.2026.800

**Published:** 2026-08-20

**Authors:** Olivier Brice BOGNING MEJIOZEM, Junior Sylvère GBELESSO, Stéphane Francia MALENDELE, Yannick Wilson HENKO KINDING, Valère NDOMA NGATCHOUKPO, Jean Chrysostome GODY

**Affiliations:** 1 Unité de néonatologie, Complexe hospitalier universitaire pédiatrique de Bangui, Faculté des sciences de la santé, Université de Bangui, République centrafricaine; 2 Unité de néonatologie, Complexe hospitalier universitaire pédiatrique de Bangui, République centrafricaine; 3 Unité de chirurgie pédiatrique, Complexe hospitalier universitaire pédiatrique de Bangui, Faculté des sciences de la santé, Université de Bangui, République centrafricaine

**Keywords:** Anomalies de fermeture du tube neural, Nouveauné, Supplémentation en acide folique, ODD 3, République centrafricaine, Afrique subsaharienne, Neural Tube Defects, Newborn, Folic acid supplementation, SDG 3, Central African Republic, Sub-Saharan Africa

## Abstract

**Introduction:**

Les anomalies de fermeture du tube neural (AFTN) représentent un indicateur sentinelle des inégalités de santé maternelle et néonatale. En République centrafricaine, l’absence d’études pédiatriques exhaustives empêche d’estimer le fardeau réel des AFTN et limite le développement de stratégies nationales de prévention efficaces, d’où l’objectif de cette étude.

**Matériel et méthode:**

Il s’agissait d’une étude rétrospective descriptive menée sur une période de 10 ans et 9 mois, de janvier 2015 à septembre 2025. Elle incluait tous les nouveaunés et nourrissons hospitalisés pour AFTN au Complexe hospitalier universitaire pédiatrique de Bangui (CHUPB), dont les dossiers étaient exploitables. Les analyses statistiques étaient réalisées à l’aide du logiciel RStudio.

**Résultats:**

116 cas d’AFTN ont été recensés, soit une fréquence hospitalière de 37 pour 10000 admissions. L’âge moyen à l’admission était de 9 jours (sexratio 1,15) et 51,7 % résidaient en zone rurale. Le suivi prénatal était insuffisant dans 95,7 % des cas et 14,7 % avaient des antécédents familiaux d’AFTN. Parmi les mères supplémentées en acide folique (n = 86), seulement 6 % avaient débuté la prise avant la conception. Le spina bifida représentait 76,7 % des cas et l’encéphalocèle 20,7 %. Une intervention chirurgicale a été réalisée chez 67,2 % des enfants. Le taux de létalité était de 33,6 % et 97 % des survivants présentaient des séquelles neurologiques.

**Conclusion:**

Les AFTN constituent un problème majeur de santé publique au CHUPB, avec mortalité et handicap élevés. Ces résultats révèlent des opportunités manquées pour l’atteinte de l’Objectif de développement durable n° 3 des Nations Unies (ODD 3), notamment la réduction de la mortalité néonatale. La prise en charge chirurgicale précoce améliore la survie, mais la prévention par supplémentation en acide folique doit être intégrée aux politiques nationales pour réduire le fardeau des AFTN.

## Abstract (en)

**Introduction:**

Neural tube closure defects (NTDs) are a sentinel indicator of maternal and neonatal health inequalities. In the Central African Republic, the lack of comprehensive pediatric studies prevents estimation of the true NTD burden and limits development of effective national prevention strategies. This study aims to address this issue.

**Materials and methods:**

This retrospective descriptive study was conducted over 10 years and 9 months, from January 2015 to September 2025. The study included all newborns and infants hospitalized for NTDs at the Bangui Pediatric University Hospital Complex (CHUPB) whose medical records were usable. Statistical analyses were performed using RStudio software.

**Results:**

A total of 116 cases of NTDs were identified, representing a hospitalization rate of 37 per 10,000 admissions. The mean age at admission was nine days (sex ratio 1.15), and 51.7% of patients lived in rural areas. Prenatal care was inadequate in 95.7% of cases, and 14.7% had a family history of NTDs. Among the mothers who took folic acid supplements (n = 86), only 6% started taking them before conception. Spina bifida accounted for 76.7% of cases and encephalocele for 20.7%. Surgical intervention was performed on 67.2% of the children. The case-fatality rate was 33.6%, and 97% of survivors experienced neurological sequelae.

**Conclusion:**

NTDs are a significant public health concern at the CHUPB, associated with high mortality and disability rates. These results reveal missed opportunities to achieve United Nations Sustainable Development Goal No. 3 (SDG 3), especially regarding the reduction of neonatal mortality. While early surgical management improves survival, prevention through folic acid supplementation must be integrated into national policies to reduce the burden of NTDs.

**Figure:**
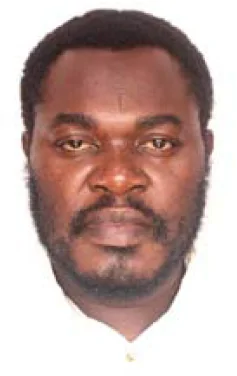

## Introduction

Les anomalies de fermeture du tube neural (AFTN) sont des malformations congénitales graves affectant le système nerveux central. Elles représentent la deuxième anomalie congénitale la plus fréquente chez l’humain [1,15]. La prévalence mondiale est estimée à 2 cas pour 1000 naissances vivantes, soit entre 214 000 et 322 000 grossesses affectées chaque année [3]. Elle est plus élevée en Afrique, atteignant jusqu’à 5,1 cas pour 1000 naissances vivantes, avec des variations régionales importantes : jusqu’à 84,5 cas pour 10 000 naissances en Afrique de l’Est et environ 10,2 pour 10 000 en Afrique centrale [5]. Les AFTN entraînent un fardeau important: environ 88000 décès néonataux chaque année et 8,6 millions d’années de vie ajustées sur l’incapacité [1,6]. Dans les pays à faible revenu, elles pourraient représenter jusqu’à 29 % des décès néonataux dus à des malformations congénitales visibles [17]. La supplémentation en acide folique pré-conceptionnelle peut prévenir jusqu’à 80 % des cas, mais l’absence de fortification ou de programmes efficaces ne permet pas de réduire de nombreux cas évitables [6].

En République centrafricaine, peu d’études pédiatriques existent, les données disponibles étant fragmentaires : une étude de 2016 sur le spina bifida et les encéphalocèles [17] et un cas d’anencéphalie publié en 2019 [16]. L’absence de données exhaustives limite l’évaluation du poids réel des AFDN et entrave la planification de stratégies nationales de prévention.

Notre travail visait à décrire le fardeau, les caractéristiques et l’évolution des anomalies de fermeture du tube neural au CHUPB, afin d’identifier les lacunes de prévention et de prise en charge et d’informer les politiques de santé maternelle et néonatale en République centrafricaine.

## Matériels et méthodes

Nous avons réalisé une étude rétrospective descriptive portant sur 10 ans et 9 mois (janvier 2015-septembre 2025) au CHUPB, unique centre de référence pour les enfants en détresse vitale en République centrafricaine. Cette étude a inclus les nouveaunés et nourrissons hospitalisés en néonatologie et chirurgie pédiatrique, admis directement depuis leur domicile ou référés par des structures de santé publiques ou privées de Bangui et des provinces (Fig. 1).

**Figure 1:**
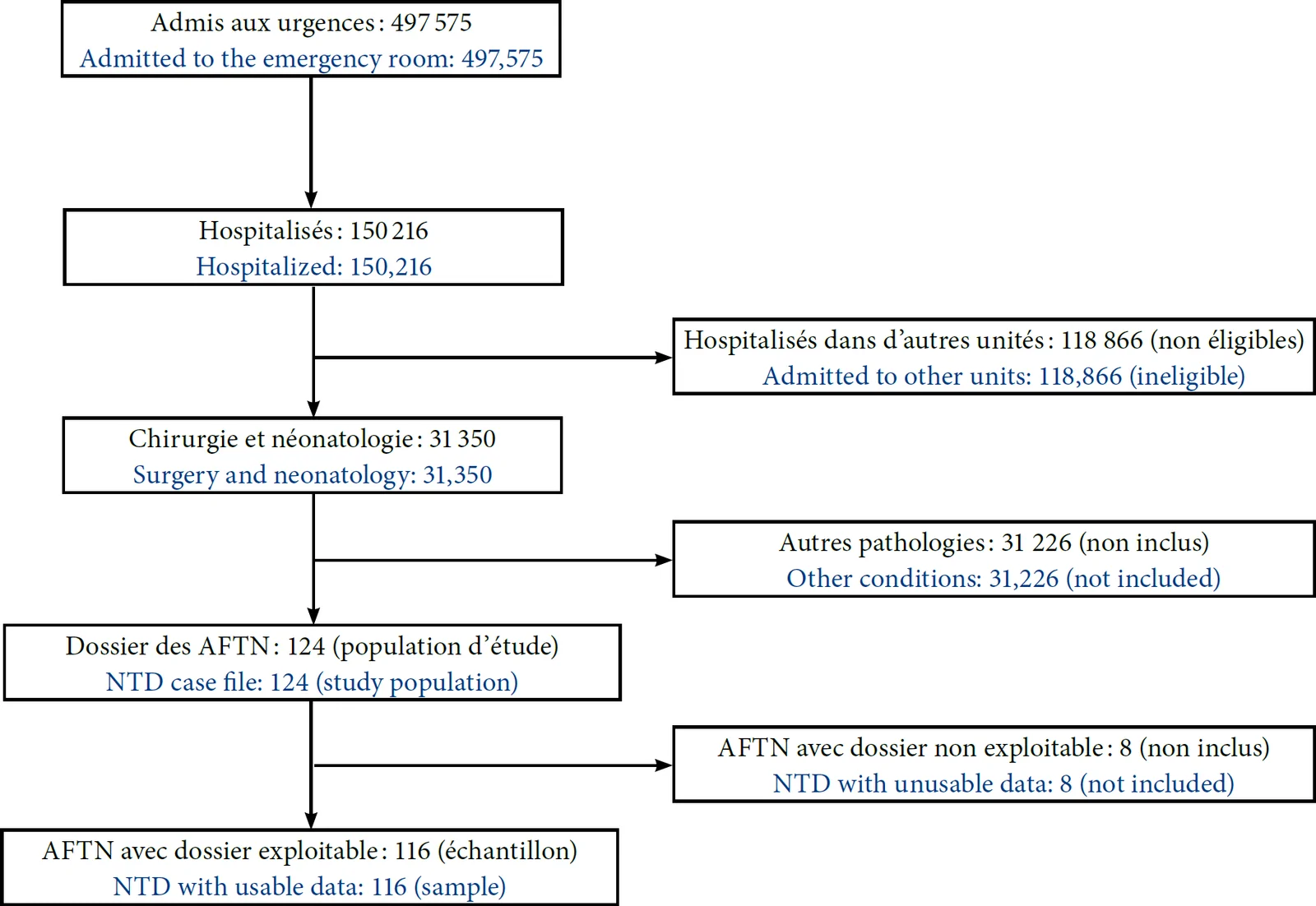
Flux de recrutement des enfants

Les dossiers retenus devaient mentionner le diagnostic « Anomalies de fermeture du tube neural » à la sortie ou au décès et être complets, avec toutes les informations nécessaires pour la fiche d’enquête. Le recueil des données a débuté après approbation du protocole par le décanat de la Faculté des sciences de la santé, et accord des chefs de département, chefs d’unité et infirmiers majors. Les registres d’hospitalisation ont été consultés pour identifier les cas, les dossiers récupérés auprès du service des archives, et les informations extraites sur une fiche préétablie, saisies dans Microsoft Excel 2016 pour nettoyage initial, puis importées dans RStudio (v.2025.05.1+513) pour analyse statistique.

Les variables étudiées comprenaient les données sociodémographiques, l’anamnèse, le suivi prénatal, la supplémentation en acide folique, les caractéristiques cliniques, la prise en charge et l’évolution des enfants. La confidentialité a été strictement respectée et tout conflit d’intérêt évité.

## Résultats

Sur 31350 enfants hospitalisés pendant la période d’étude, 116 cas d’anomalies du tube neural ont été colligés, correspondant à une fréquence hospitalière de 37 pour 10000 admissions. La répartition des enfants selon les années d’inclusion est présentée à la Figure 2.

**Figure 2:**
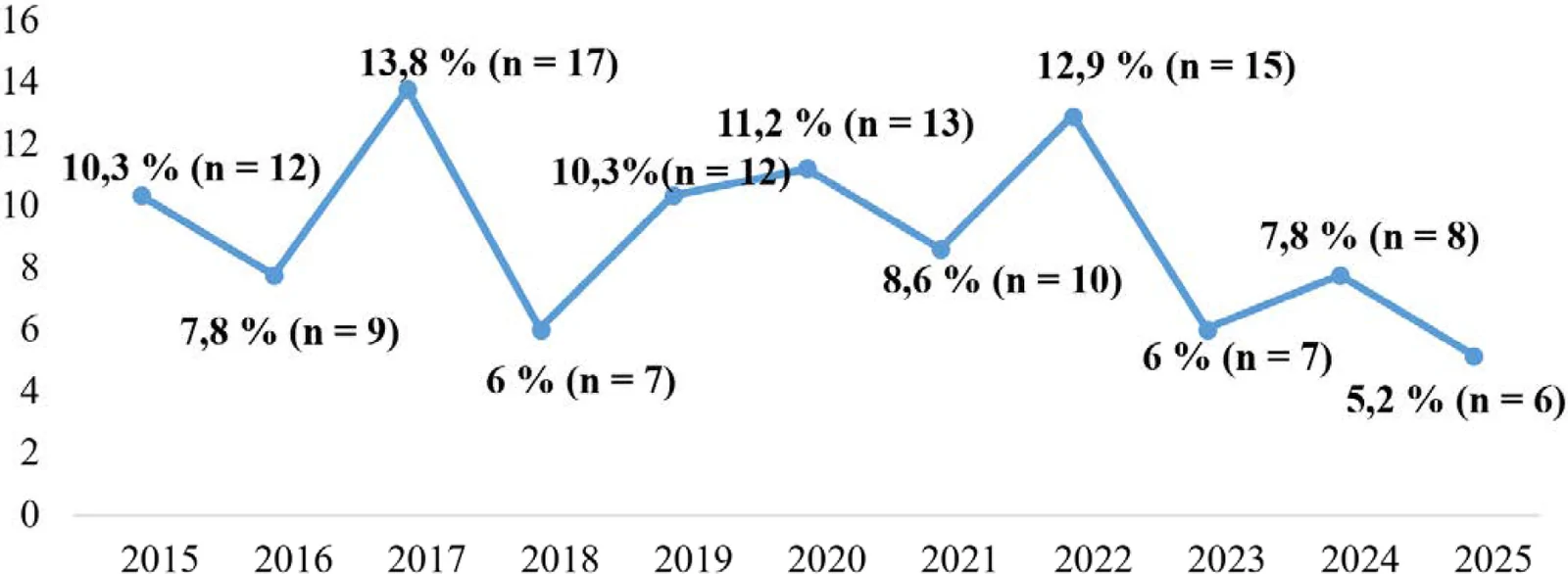
Répartition des enfants en fonction des années d’inclusion (N = 116)

Les enfants étaient répartis en 53,4 % (n = 62) de garçons et 46,6 % (n = 54) de filles, soit un sexratio de 1,15. À l’admission, 85 % (n = 99) des enfants étaient en période néonatale et 15 % (n = 17) en période postnatale. L’âge moyen était de 9 ± 8 jours (extrêmes: 0-30 jours) et la médiane de 3,5 jours. Parmi les enfants inclus, 60 (51,7 %) résidaient en zone rurale et 56 (48,3 %) en zone urbaine. Le mode d’admission était le suivant : 58 (50 %) par évacuation depuis les provinces, 51 (44 %) par référence des maternités de Bangui et 7 (6 %) admissions directes depuis le domicile.

Dixsept enfants (14,7 %) présentaient des antécédents familiaux d’AFTN. Un suivi prénatal conforme aux recommandations de l’OMS était rapporté dans 4,3 % des cas (n = 5). Au moins une échographie obstétricale avait été réalisée chez 31 % (n = 36) des mères.

Les mères avaient reçu une supplémentation en acide folique dans 74,1 % des cas (n = 86). Parmi elles, seules cinq (5,8 %) l’avaient débutée avant la conception et 94,2 % (n = 81) en période péri-conceptionnelle.

Sur les 81 mères ayant pris de l’acide folique en période péri-conceptionnelle, 34,6 % (n = 28) avaient débuté la prise entre la 6^e^ et la 12^e^ se-maine d’aménorrhée, 43,2 % (n = 35) après la 12^e^ et jusqu’à la 18^e^ semaine d’aménorrhée et le reste après la 18^e^ semaine d’aménorrhée.

Parmi les 86 mères supplémentées en acide folique, la prescription était effectuée par des sages-femmes dans 52 cas (60,5 %), par des médecins généralistes dans 17 cas (19,8 %), par des gynécologues dans 10 cas (11,6 %) et par des assistantes-accoucheuses dans 7 cas (8,1 %).

Le poids moyen à l’admission était de 2 815 g (extrêmes : 1 000-4 010 g). La répartition des enfants selon le poids à l’admission est présentée à la Figure 3.

**Figure 3:**
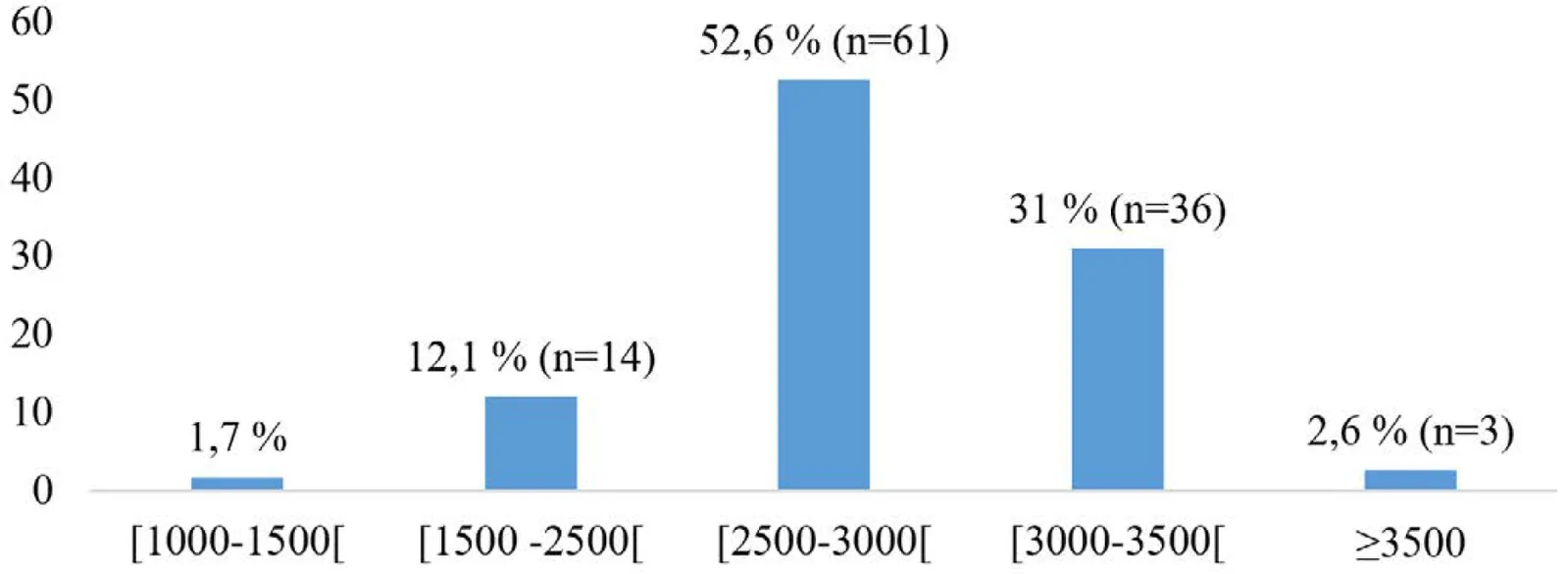
Répartition des enfants selon le poids à l’admission)

Parmi les 116 enfants inclus, 89 (76,7 %) pré-sentaient un spina bifida, 24 (20,7 %) une encé-phalocèle, 2 (1,7 %) une myéloméningocèle et 1 (0,9 %) une anencéphalie.

Dans notre série 67,2 % (n = 78) des cas avaient bénéficié d’une intervention chirurgicale, pour 32,8 % (n = 38) qui n’avaient pas été opérés.

L’évolution à court terme était favorable pour 68 enfants (56,9 %), 9 (7,8 %) étaient sortis contre avis médical, et le taux de létalité était de 33,6 % (n = 39).

Parmi les 78 nourrissons opérés, 92 % (n = 72) des interventions étaient réalisées entre le 7e et le 48e jour de vie, avec un délai moyen d’intervention chirurgicale de 26 ± 12 jours et des extrêmes de 5 à 60 jours de vie. Dans ce groupe, 13 enfants (17 %) étaient décédés et 65 (83 %) avaient survécu. Parmi les 38 patients non opérés, 68 % (n = 26) étaient décédés, 8 % (n = 3) avaient survécu et 247 % (n = 9) étaient sortis contre avis médical. Les 68 enfants survivants présentaient des sé-quelles neurologiques dans 97 % (n = 66) des cas, tandis que 3 % (n = 2) n’en avaient pas. La durée moyenne de séjour était de 12,5 jours avec des extrêmes de 1 à 59 jours.

## Discussion

Les AFTN constituent un indicateur sentinelle des inégalités de santé maternelle et néonatale à l’échelle mondiale [29]. Leur prévalence varie considérablement selon les contextes géogra-phiques, le niveau de développement des systèmes de santé et les stratégies de prévention mises en œuvre, notamment la supplémentation et la fortification en acide folique [2,29]. Dans les pays à revenu faible et intermédiaire, comme la République centrafricaine, l’absence de registres nationaux des malformations congénitales et de systèmes de notification systématique des cas, en particulier ceux survenant en maternité ou aboutissant à des décès périnataux précoces, rend difficile l’estimation réelle de la prévalence des AFTN [4]. Les données hospitalières disponibles sousestiment ainsi fréquemment leur poids réel et peuvent introduire un biais de sélection important [4].

Dans notre série, la fréquence hospitalière des AFTN était de 37 cas pour 10000 admissions au CHUPB. Ce chiffre demeure inférieur à celui rapporté dans certaines études hospitalières menées en Afrique centrale et orientale, notamment en République démocratique du Congo et en Éthiopie, où des incidences comprises entre 57 et 250 pour 10 000 naissances vivantes ont été décrites [2,23]. De manière générale, dans les pays à faible revenu, la prévalence hospitalière des AFTN varie entre 3 et 8,7 cas pour 10 000 naissances, avec une forte hétérogénéité selon les régions et les méthodologies utilisées [2,29]. À l’inverse, dans les pays à revenu élevé, la prévalence est mieux documentée grâce à des systèmes de surveillance performants. En Europe, elle est estimée autour de 10 cas pour 10 000 naissances [14], tandis qu’aux États-Unis, elle varie entre 1,5 et 3,5 cas pour 10 000 naissances vivantes [24,3]. Plusieurs facteurs peuvent expliquer la fréquence hospitalière relativement élevée observée dans notre contexte. La pauvreté structurelle, exacerbée par des crises socio-militaro-politiques répétées, favorise des conditions de vie précaires et un déficit nutritionnel, notamment en acide folique, reconnu comme facteur de risque majeur des AFTN [13]. Par ailleurs, la faible couverture sanitaire et l’insuffisance du suivi prénatal, observées chez 85 % des mères dans notre série, limitent à la fois la prévention pré-conceptionnelle et le dépistage anténatal des malformations [20]. L’absence de politique nationale de fortification des aliments de base en acide folique et de programmes de supplémentation ciblant les femmes en âge de procréer contribue également à la persistance d’un nombre élevé de cas évitables [11,22]. Enfin, la comparaison entre les données hospitalières et l’incidence réelle en population générale demeure délicate, car les études hospitalières n’incluent que les cas admis et excluent les malformations non diagnostiquées ou les décès périnataux non enregistrés, ce qui limite la représentativité des résultats [13].

La prévention des AFTN repose sur une supplé-mentation en acide folique avant la conception et au début de la grossesse [2,13,20]. Dans notre série, moins de 6 % des mères avaient commencé la supplémentation en période pré-conceptionnelle, alors que plus des deux tiers avaient reçu de l’acide folique pendant la grossesse, illustrant un échec des stratégies actuelles centrées sur la grossesse confirmée [11,22]. La fortification obligatoire des aliments de base reste l’intervention la plus efficace et équitable, permettant une diminution de 70 à 80 % des AFTN dans plusieurs pays [10,8]. L’absence de cette politique en République centrafricaine expose les femmes, en particulier en milieu rural ou en situation de précarité, à un risque élevé de malformations évitables. Dans ce contexte, nos résultats fournissent des arguments solides en faveur de l’intégration de la fortification en acide folique comme priorité de santé publique nationale.

Le suivi prénatal non conforme aux recommandations, observé chez plus de 98 % (n = 114) des mères reflète une couverture sanitaire insuffisante et des inégalités marquées d’accès aux services de santé maternelle [19,25]. Cette situation limite non seulement la prévention par acide folique, mais également le dépistage anténatal des malformations congénitales et l’orientation précoce vers des structures spécialisées. Dans les pays à revenu élevé, le dépistage prénatal permet d’organiser la prise en charge périnatale, d’optimiser la planification chirurgicale et, dans certains contextes, d’envisager des options thérapeutiques ou de conseil adaptées [9,15,27]. L’absence de ces dispositifs en République centrafricaine contribue à des admissions tardives, souvent dans un état clinique déjà critique, avec un impact direct sur la mortalité et les séquelles à long terme.

L’âge moyen à l’admission de neuf jours met en évidence des retards majeurs dans le référencement des nouveaunés porteurs d’AFTN. Ces délais reflètent des failles dans l’organisation du système de santé, notamment l’absence de réseaux de référencement fonctionnels, la dépendance à des moyens de transport non médicalisés et le manque de sensibilisation des communautés et des professionnels de santé de première ligne [21]. La chirurgie néonatale précoce constitue un déterminant clé de la survie et du pronostic fonctionnel des enfants atteints d’AFTN [7,15]. Dans notre série, près d’un tiers des enfants n’a pas pu bénéficier d’une intervention chirurgicale, principalement en raison de contraintes logistiques, financières et humaines. Le renforcement des capacités chirur-gicales pédiatriques et anesthésiques, ainsi que l’intégration des soins néonataux spécialisés dans les politiques nationales de santé, apparaissent indispensables pour réduire la mortalité évitable. La mortalité observée (33,6 %) et la proportion extrêmement élevée (plus de 97 %) de séquelles neurologiques chez les survivants (n = 66), illustre le lourd fardeau humain, social et économique des AFTN dans notre série. Audelà de la survie néonatale, les enfants atteints nécessitent souvent des soins à long terme, une rééducation spécialisée et un accompagnement social, rarement disponibles dans les pays à faible revenu comme la République centrafricaine [7,12,18].

Les conséquences s’étendent ainsi aux familles et aux communautés, renforçant les cycles de pauvreté et d’exclusion. Dans une perspective de santé publique, pour tous les pays, y compris la République centrafricaine, le taux de létalité très élevé et les séquelles quasi systématiques chez les survivants d’AFTN observés dans notre série représentent un obstacle majeur à l’atteinte des Objectifs de développement durable, en particulier l’ODD 3 qui vise à réduire la mortalité néonatale et à améliorer la santé maternelle [26]. La prévention des AFTN par des interventions simples et peu coûteuses, telles que la fortification en acide folique, représente l’un des investissements les plus rentables en santé maternelle et infantile [5,11,28]. Cette étude rétrospective reposait sur l’exploitation de données hospitalières, ce qui limite les paramètres analysés aux informations disponibles dans les dossiers et peut affecter la qualité des données. Elle a porté sur les AFTN dans l’unité de néonatologie et le service de chirurgie du CHUPB, unique centre de référence national, qui prend en charge environ 95 % des nouveaunés et nour-rissons présentant des malformations graves en République centrafricaine et dans la zone voisine du Congo. La taille de l’échantillon et la durée de collecte sur 10 ans et 9 mois permettent une analyse statistique robuste, et les données obtenues constituent une base hospitalière de référence pour de futures études en collaboration avec d’autres hôpitaux et services de maternité du pays.

## Conclusion

Cette étude met en évidence des lacunes majeures dans le suivi prénatal, la supplémentation en acide folique et l’accès précoce à la chirurgie des anomalies de fermeture du tube neural en République centrafricaine. Bien que l’intervention chirurgicale améliore la survie, la majorité des nouveaunés et nourrissons présente encore des séquelles neurologiques.

Au regard des résultats obtenus, il apparaît nécessaire de renforcer les stratégies de prévention et de prise en charge de ces anomalies dans ce pays. Sur le plan de la prévention primaire, les autorités sanitaires devraient promouvoir des mesures minimales de santé maternelle, notamment la supplémentation systématique en acide folique et le renforcement des conseils nutritionnels destinés aux femmes en âge de procréer.

Sur le plan du dépistage prénatal, la mise à disposition d’équipements d’échographie dans l’ensemble des formations sanitaires, associée à la gratuité de l’échographie obstétricale conformément aux recommandations de la politique nationale de santé, permettrait de systématiser son intégration dans le suivi prénatal et d’améliorer la détection précoce des malformations fœtales.

Enfin, sur le plan de la prise en charge, il est recommandé aux autorités sanitaires d’optimiser le circuit de référence afin de permettre une intervention chirurgicale plus précoce en période néonatale. Cela implique le renforcement des structures périphériques, la formation du personnel de santé et la mise en place d’un réseau fonctionnel de suivi et d’orientation materno-néonatale. La gratuité ciblée des soins pour les femmes enceintes et les enfants de moins de cinq ans contribuerait également à améliorer l’accès aux soins spécialisés, en cohérence avec l’ODD 3 relatif à la santé et au bienêtre.

## Approbation éthique

Cette étude a été approuvée par le décanat de la Faculté des sciences de la santé (Université de Bangui). Le protocole a été évalué et validé par les chefs de départements, chefs d’unités et infirmiers-majors du Complexe hospitalier universitaire pédiatrique de Bangui. Le respect de la confidentialité des patients a été strictement assuré. Le consentement éclairé des parents ou tuteurs légaux a été obtenu pour l’utilisation des images médicales.

## Financement

Aucun financement spécifique n’a été reçu pour la réalisation de cette étude. Les auteurs déclarent que la réalisation de ce manuscrit n’a bénéficié d’aucune subvention ni soutien institutionnel ou commercial.

## Contributions des auteurs

BOGNING MEJIOZEM Olivier Brice: rédaction du manuscrit, supervision générale de l’étude

GBELESSO Junior Sylvère : collecte des données cliniques et suivi des interventions

MALENDELE Stéphane Francia: suivi néonatal et collecte des données sur les nourrissons

HENKO KINDING Yannick Wilson : analyse des données et suivi post-opératoire

NDOMA NGATCHOUKPO Valère: supervision chirurgicale et validation des données opératoires

GODY Jean Chrysostome: supervision, validation de l’étude et supervision de la version finale

## Déclaration de liens d’intérêt

Aucun lien d’intérêt n’a été déclaré.

## Figures and Tables

**Figure A1:**
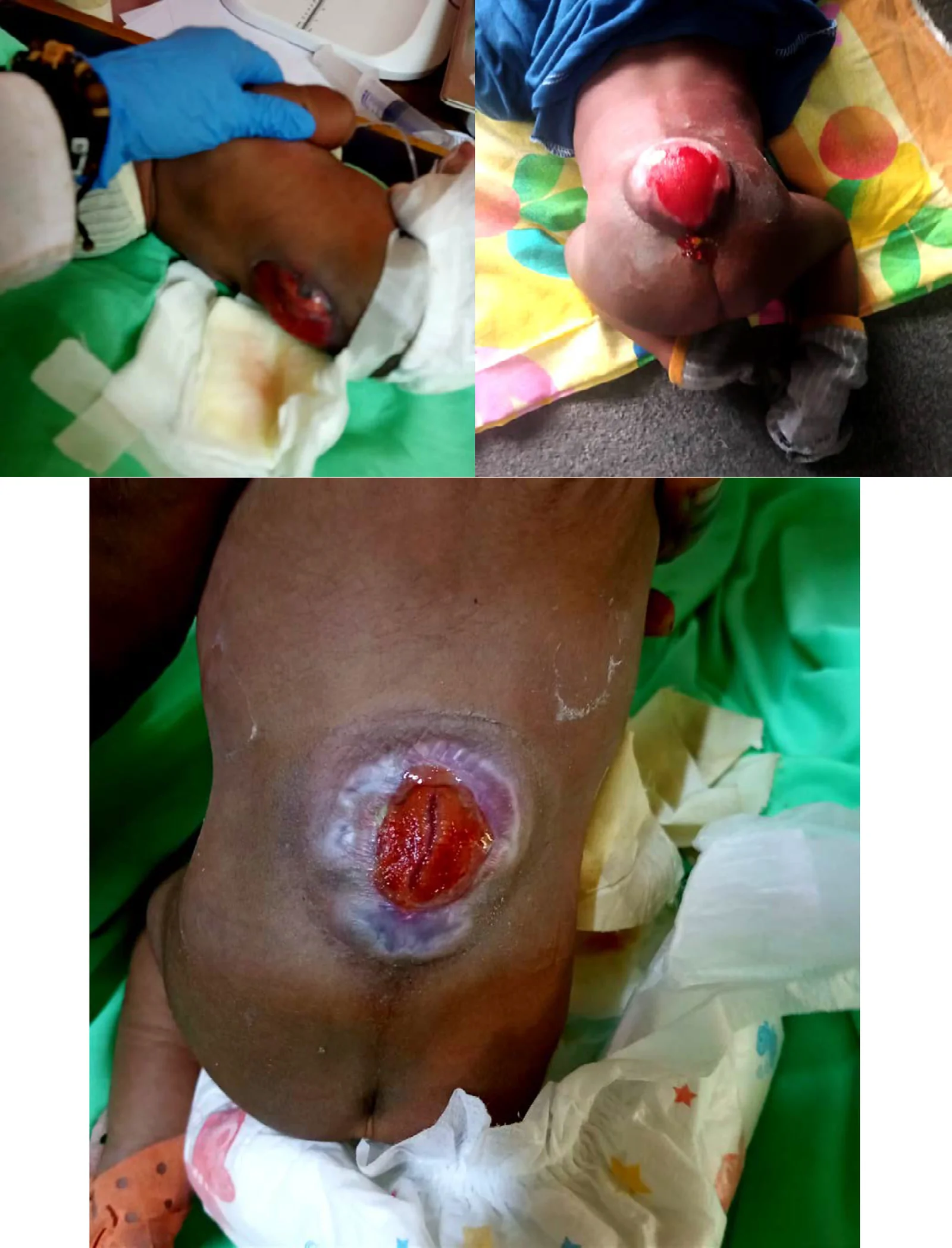
Spina bifida lombaire avec sac méningé infecté, CHUPB

**Figure A2:**
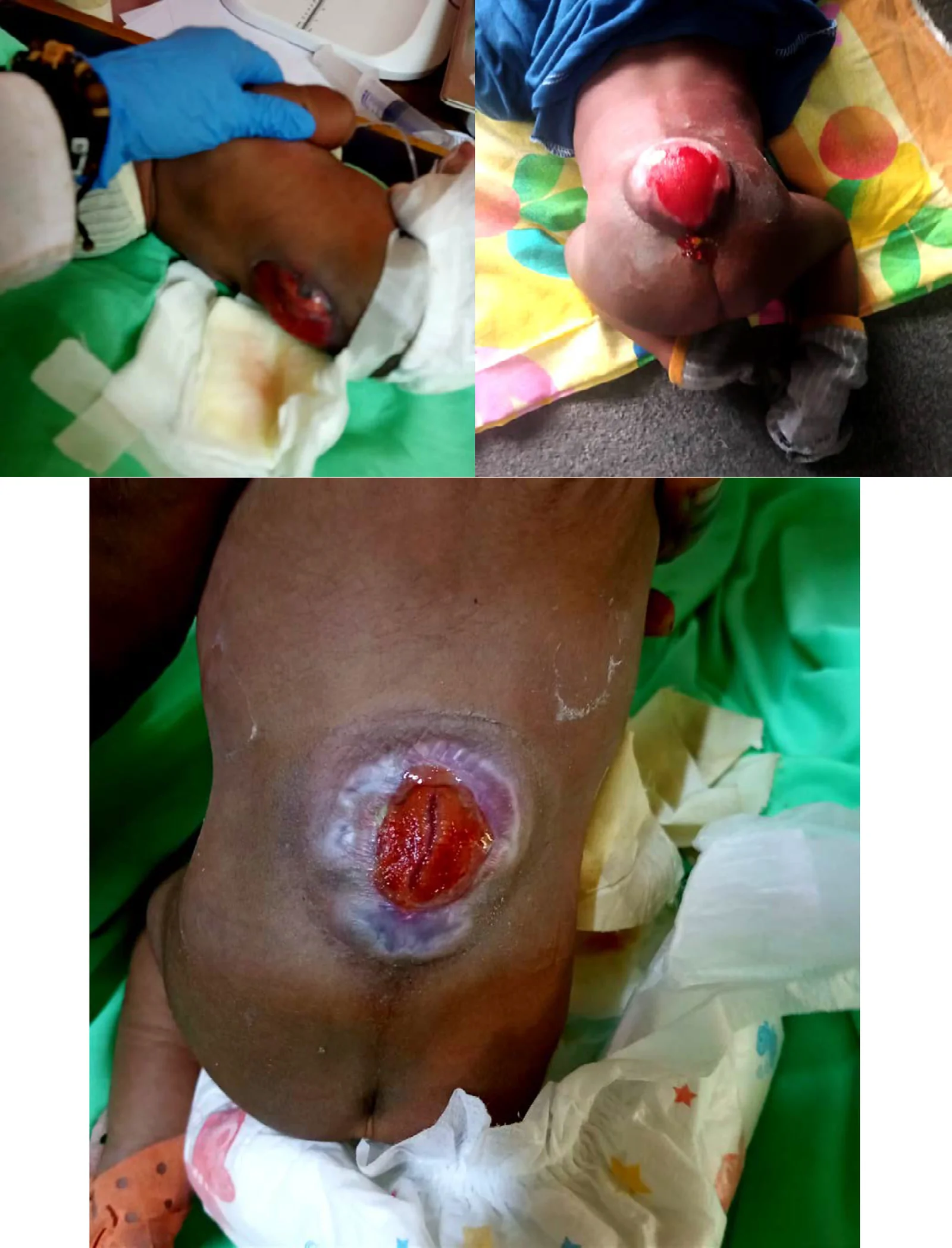
Spina bifida lombaire avec sac méningé non infecté, CHUPB

**Figure 3A:**
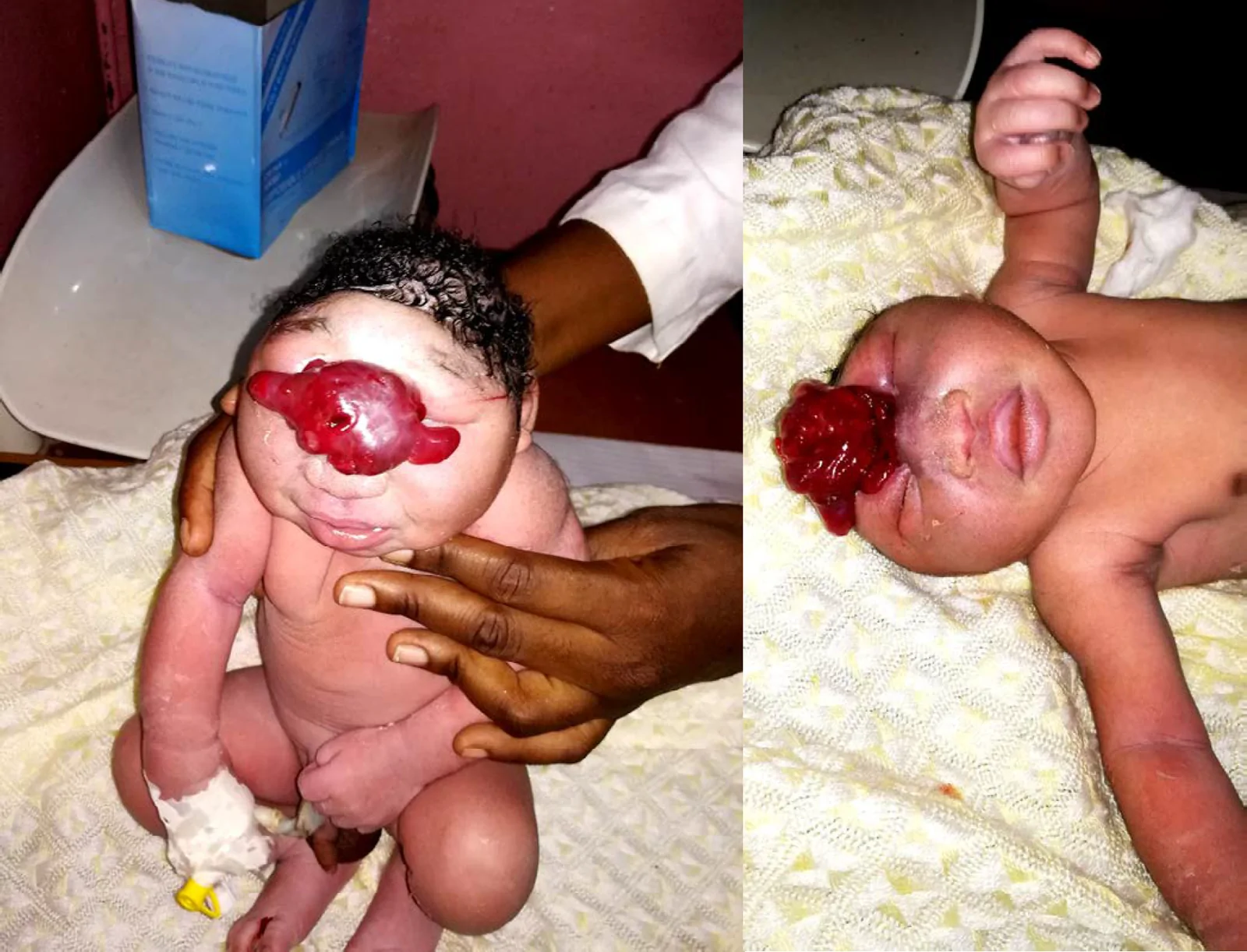
Encéphalocèle fronto-nasal, CHUPB

**Figure 4A:**
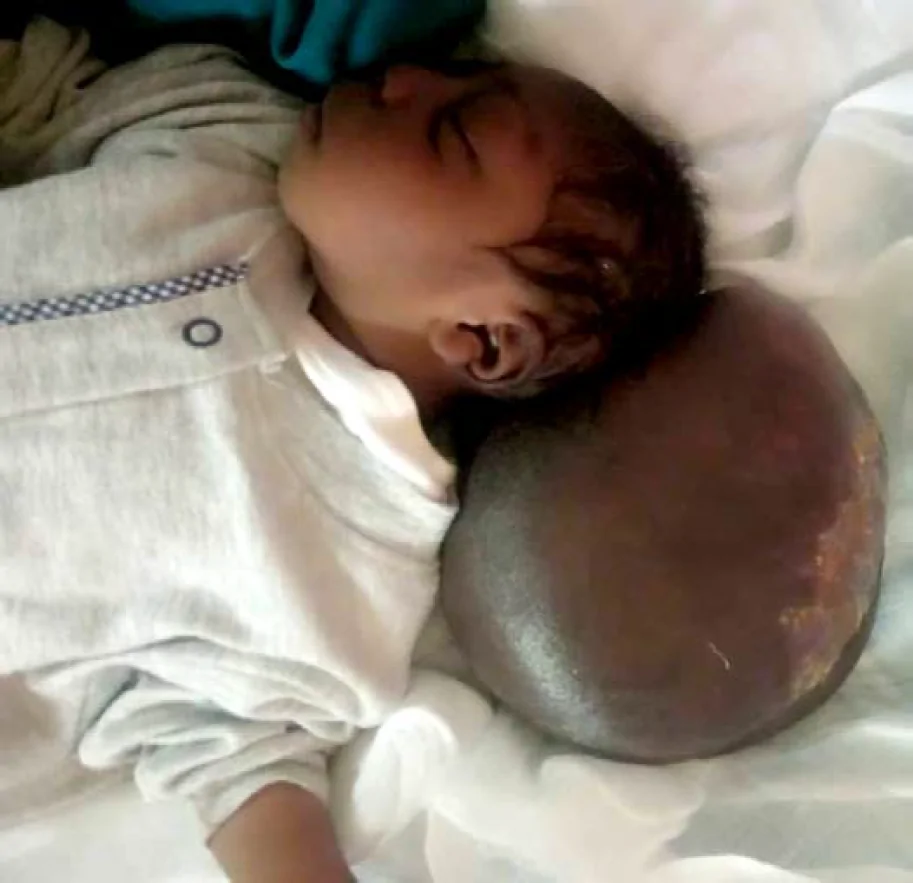
Encéphalocèle occipital, CHUPB

**Figure 5A:**
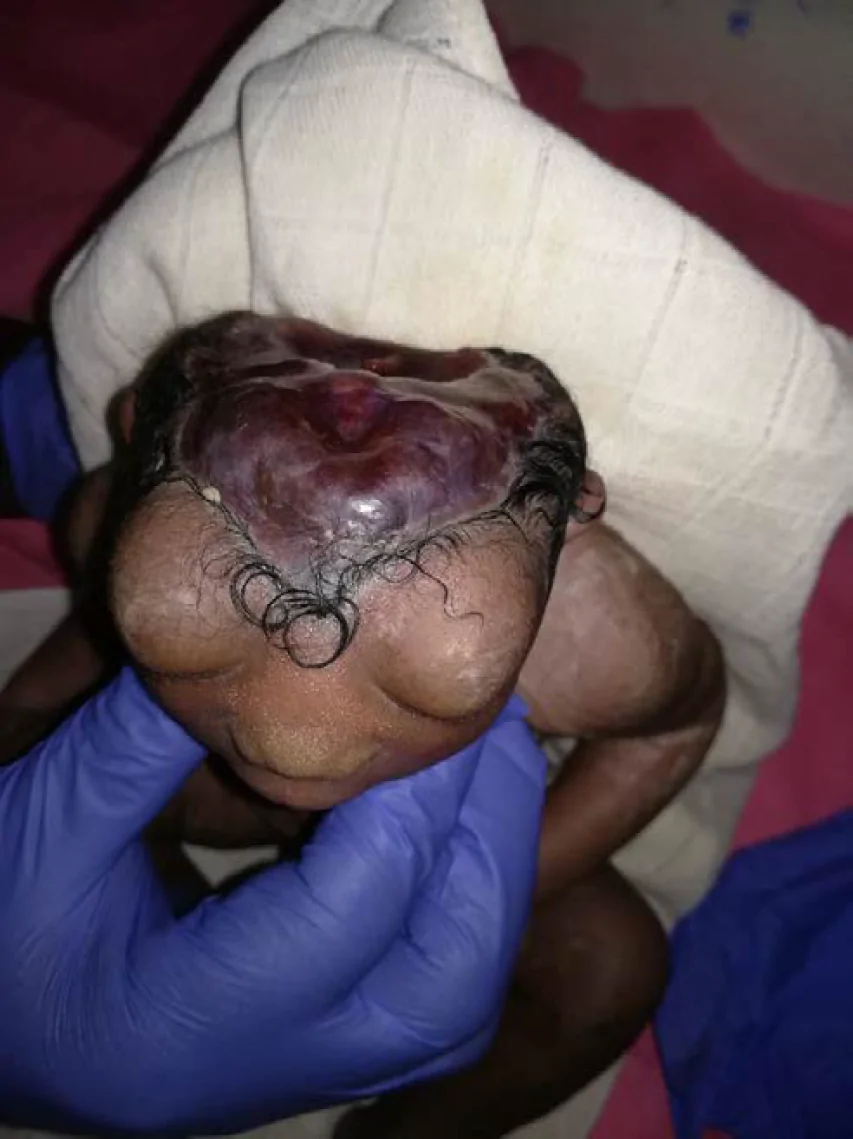
Anencéphalie, CHUPB

**Figure 6A:**
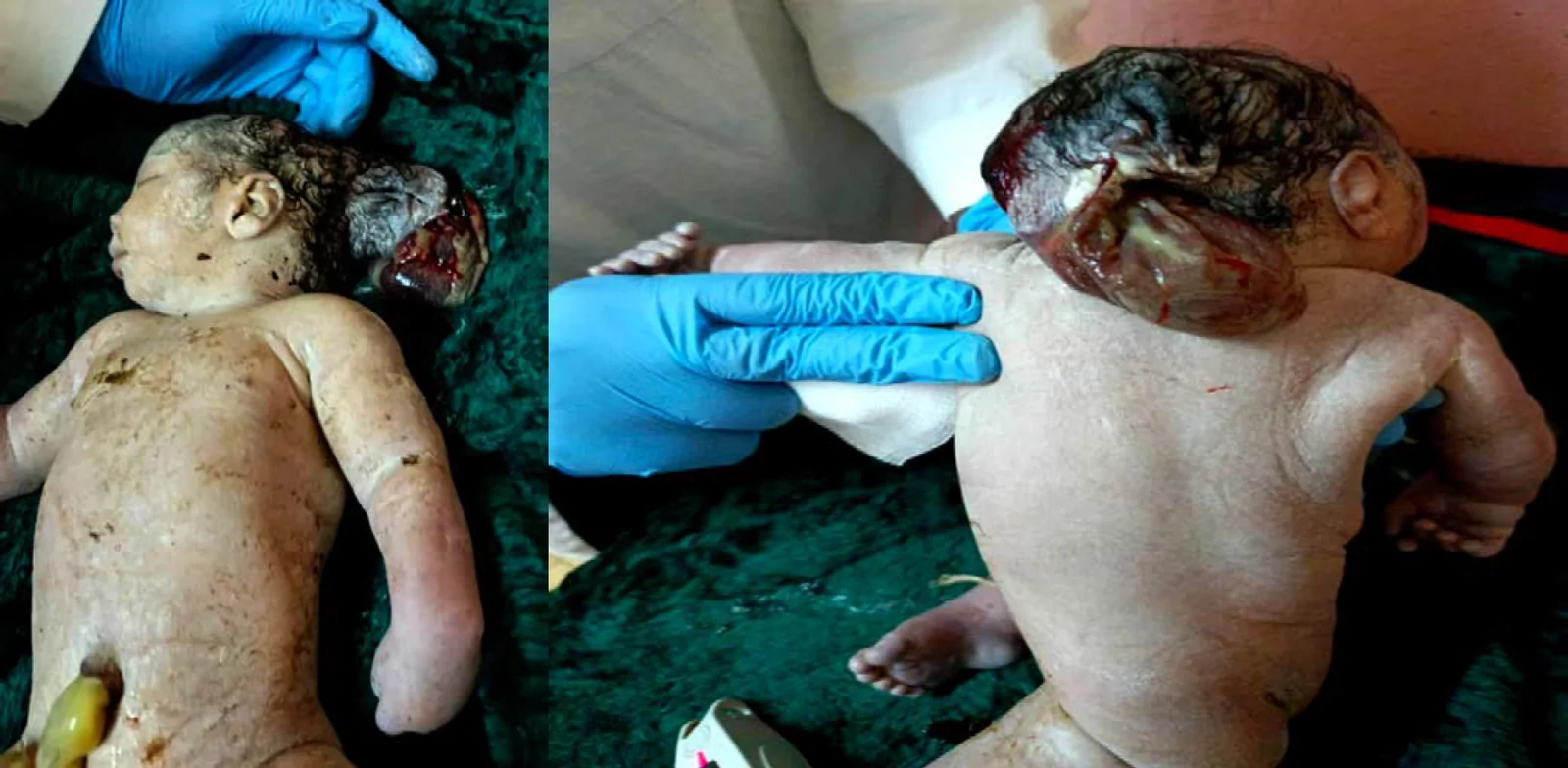
Encéphalocèle occipital rompu, CHUPB
