# Supplementary material for: Neural tube closure defects in the Central African Republic: high mortality and missed opportunities for SDG 3
Source: Med Trop Sante Int. 2026 Aug 20;6(3):mtsi.v6i3.2026.800. [Article in French] doi: 10.48327/mtsi.v6i3.2026.800 (PMC13697907; doi:10.48327/mtsi.v6i3.2026.800)
Supplement: Related texts

# Research article: Neural tube closure defects in the Central African Republic: high mortality and missed opportunities for SDG 3

## Introduction

Neural tube closure defects (NTDs) are serious congenital malformations that affect the central nervous system. They are the second most common congenital anomaly in humans [1,15]. The global prevalence is estimated at two cases per 1,000 live births, representing 214,000 to 322,000 affected pregnancies annually [3]. Prevalence is higher in Africa, reaching up to 5.1 cases per 1,000 live births. There are significant regional variations, with up to 84.5 cases per 10,000 births in East Africa and approximately 10.2 per 10,000 in Central Africa [5]. NTDs impose a significant burden, causing approximately 88,000 neonatal deaths and 8.6 million disability-adjusted life years each year [1,6]. In low-income countries, NTDs may account for up to 29% of neonatal deaths due to visible congenital malformations [17]. Preconception folic acid supplementation can prevent up to 80% of cases; however, the lack of fortification or effective programs prevents reducing many preventable cases [6].

In the Central African Republic, few pediatric studies exist. The available data is limited to a 2016 study on spina bifida and encephaloceles [17] and a 2019 case report on anencephaly [16]. The lack of comprehensive data hinders the assessment of the true burden of NTDs and the planning of national prevention strategies.

Our study aimed to describe the characteristics and trends of neural tube closure defects at CHUPB in order to identify gaps in prevention and care and inform maternal and neonatal health policies in the Central African Republic.

## Materials and methods

We conducted a retrospective descriptive study at the CHUPB, the sole referral center for critically ill children in the Central African Republic, covering a period of 10 years and 9 months (January 2015–September 2025). The study population included newborns and infants hospitalized in the neonatology and pediatric surgery units who were admitted directly from their homes or referred by public or private health facilities in Bangui and the provinces (Fig. 1).

**Figure 1:** Child enrollment flowchart

Selected records had to include a diagnosis of “neural tube closure defects” at discharge or death and be complete with all the necessary information for the survey form. Data collection began after the protocol was approved by the Dean’s Office of the Faculty of Health Sciences and department, unit, and head nurse heads consented. We reviewed hospital admission records to identify cases, retrieved medical records from the archives department, and entered the information onto a pre-designed form. We then imported the data into Microsoft Excel 2016 for initial cleaning and into RStudio (v.2025.05.1+513) for statistical analysis.

The variables studied included sociodemographic data, medical history, prenatal care, folic acid supplementation, clinical characteristics, management, and the children’s outcomes. Confidentiality was strictly maintained, and any conflicts of interest were avoided.

## Results

During the study period, 31,350 children were hospitalized. Of those, 116 cases of neural tube defects were identified, corresponding to a hospital incidence rate of 37 per 10,000 admissions. Figure 2 shows the distribution of children by year of inclusion.

**Figure 2:** Distribution of children by years of inclusion (N = 116)

Of these children, 53.4% (n = 62) were boys and 46.6% (n = 54) were girls, resulting in a sex ratio of 1.15. At admission, 85% (n = 99) of the children were in the neonatal period, while the remaining 15% (n = 17) were in the postnatal period. The mean age was 9 ± 8 days (range, 0–30 days), and the median age was 3.5 days.

Of the included children, 60 (51.7%) lived in rural areas, while 56 (48.3%) lived in urban areas. The mode of admission is as follows: Fifty-eight (50%) were evacuated from the provinces, 51 (44%) were referred from maternity wards in Bangui, and seven (6%) were admitted directly from home. Seventeen children (14.7%) had a family history of NTD. Prenatal care in accordance with WHO recommendations was reported in only 4.3% of cases (n = 5). At least one obstetric ultrasound was performed on 31% (n = 36) of the mothers. Folic acid supplementation was received by 74.1% of the mothers (n = 86). Of those, only five (5.8%) started taking it before conception; 94.2% (n = 81) started during the periconceptional period.

Of the 81 mothers who took folic acid during the periconceptional period, 34.6% (n = 28) started between the sixth and twelfth weeks of amenorrhea, 43.2% (n = 35) started between the thirteenth and eighteenth weeks of amenorrhea, and the remainder started after the eighteenth week of amenorrhea.

Of the 86 mothers who received folic acid supplementation, midwives issued the prescription in 52 cases (60.5%), general practitioners in 17 cases (19.8%), gynecologists in 10 cases (11.6%), and assistant midwives in seven cases (8.1%).

The mean birth weight was 2,815 g (range: 1,000–4,010 g). Figure 3 shows the distribution of children by birth weight.

**Figure 3:** Distribution of children by weight at admission

Of the 116 children included in the study, 89 (76.7%) had spina bifida, 24 (20.7%) had an encephalocele, two (1.7%) had a myelomeningocele, and one (0.9%) had an anencephaly.

Of the cases in our series, 67.2% (n = 78) had undergone surgery, while 32.8% (n = 38) had not. The short-term outcome was favorable for 68 children (56.9%). Nine children (7.8%) were dis-charged against medical advice, and the case-fatality rate was 33.6% (n = 39).

Of the 78 infants who underwent surgery, 92% (n = 72) of the procedures were performed between the seventh and 48th day of life, with a mean time to surgery of 26 ± 12 days, ranging from five to 60 days. In this group, 13 infants (17%) died, and 65 (83%) survived.

Among the 38 patients who did not undergo surgery, 68% (n = 26) died, 8% (n = 3) survived, and 24% (n = 9) were discharged against medical advice.

Of the 68 surviving children, 97% (n = 66) experienced neurological sequelae, while 3% (n = 2) did not. The mean length of stay was 12.5 days, ranging from one to 59 days.

## Discussion

NTDs serve as a sentinel indicator of maternal and neonatal health inequalities worldwide [29]. Their prevalence varies considerably depending on the geographic context, the level of development of health systems, and the implemented prevention strategies, particularly folic acid supplementation and fortification [2,29]. In low- and middle-income countries, such as the Central African Republic, the lack of national registries of congenital malformations and systematic case reporting systems, particularly for cases occurring in maternity wards or resulting in early perinatal deaths, makes it difficult to accurately estimate the prevalence of NTD [4]. Available hospital data thus frequently underestimates the actual burden of NTDs and may introduce significant selection bias [4].

In our study, the incidence rate of NTD at the CHUPB was 37 cases per 10,000 hospital admissions. This figure is lower than those reported in some hospital-based studies in Central and Eastern Africa, especially in the Democratic Republic of the Congo and Ethiopia. In these regions, incidence rates ranging from 57 to 250 cases per 10,000 live births have been documented [2,23]. In general, the hospital prevalence of NTDs in low-income countries ranges from 3 to 8.7 cases per 10,000 births, depending on the region and methodologies used [2,29]. In high-income countries, however, prevalence is better documented thanks to effective surveillance systems. In Europe, the prevalence is estimated at around 10 cases per 10,000 births [14], while in the United States, the prevalence ranges from 1.5 to 3.5 cases per 10,000 live births [24,3].

Several factors may explain the relatively high hospital admission rate observed in our setting. Structural poverty, exacerbated by repeated socio-military-political crises, contributes to precarious living conditions and nutritional deficiencies, particularly folate deficiency, which is recognized as a major risk factor for NTDs [13]. Furthermore, inadequate prenatal care and low health cover-age—experienced by 85% of the mothers in our study group—limit preconception prevention and prenatal screening for birth defects [20]. The absence of national policies to fortify staple foods with folic acid and of supplementation programs targeting women of childbearing age contributes to the persistence of a high number of preventable cases [11,22]. Finally, it is challenging to compare hospital data with the actual incidence in the general population because hospital-based studies include only admitted cases and exclude undiagnosed malformations or unrecorded perinatal deaths, which limits the representativeness of the results [13].

The prevention of NTDs relies on folic acid supplementation before and early in pregnancy [2,13,20]. In our study, fewer than 6% of mothers began taking folic acid supplements during the preconception period. In contrast, more than two-thirds took folic acid supplements during pregnancy. This illustrates the failure of current strategies that focus on confirmed pregnancies [11,22]. Mandatory fortification of staple foods is the most effective and equitable intervention, reducing NTDs by 70–80% in several countries [10,8]. The absence of such a policy in the Central African Republic exposes women, particularly those in rural areas or precarious situations, to a high risk of preventable birth defects. In this context, our findings strongly support incorporating folic acid fortification as a national public health priority.

The failure to follow prenatal care recommendations, observed in over 98% of mothers (n = 114), reflects inadequate health coverage and significant disparities in access to maternal health services [19,25]. This situation limits not only folic acid prevention, but also prenatal screening for congenital malformations and early referral to specialized facilities. In high-income countries, prenatal screening enables the organization of perinatal care, optimizes surgical planning, and allows for the consideration of appropriate treatment or counseling options in certain contexts [9,15,27]. The absence of these systems in the Central African Republic results in delayed admissions, often when patients are already in critical condition. This has a direct impact on mortality and long-term sequelae.

The average age of admission, which is nine days, highlights major delays in referring newborns with NTDs. These delays reflect flaws in the healthcare system’s organization, including the absence of functional referral networks, reliance on non-medical transportation, and a lack of awareness among communities and primary care health professionals [21].

Early neonatal surgery is a key determinant of survival and functional prognosis for children with NTDs [7,15]. In our study, nearly one-third of the children were unable to undergo surgery due to logistical, financial, and human resource constraints. Strengthening pediatric surgical and anesthesiology capacities and integrating specialized neonatal care into national health policies appears essential to reducing preventable mortality.

The observed mortality rate of 33.6% and the extremely high proportion of over 97% of neurological sequelae among survivors illustrate the heavy human, social, and economic burden of NTDs in our study. Beyond neonatal survival, affected children often require long-term care, specialized rehabilitation, and social support, which are rarely available in low-income countries such as the Central African Republic [7,12,18]. The consequences extend to families and communities as well, reinforcing cycles of poverty and exclusion. From a public health perspective, the very high case-fatality rate and near-universal long-term sequelae among NTD survivors observed in our series represent a major obstacle to achieving the Sustainable Development Goals (SDGs), particularly SDG 3, which aims to reduce neonatal mortality and improve maternal health [26]. Preventing NTDs through simple, low-cost interventions such as folic acid fortification is one of the most cost-effective investments in maternal and child health [5,11,28].

This retrospective study relied on the analysis of hospital data. This limits the analyzed parameters to the available information in the medical records, which may affect the quality of the data. The study focused on NTDs in the neonatal unit and surgical department of the CHUPB, the sole national referral center that treats approximately 95% of newborns and infants with severe malformations in the Central African Republic and the neighboring region of the Congo. The sample size and 10-year, 9-month data collection period enable robust statistical analysis. The obtained data constitute a hospital reference database for future studies in collaboration with other hospitals and maternity wards in the country.

## Conclusion

This study highlights major gaps in prenatal care, folic acid supplementation, and early access to surgery for neural tube defects in the Central African Republic. Although surgical intervention improves survival, most newborns and infants still experience neurological complications.

In light of these findings, strengthening strategies for the prevention and management of these anomalies in this country appears necessary.

In terms of primary prevention, health authorities should promote basic maternal health measures, including routine folic acid supplementation and enhanced nutritional counseling for women of childbearing age.

Regarding prenatal screening, making ultrasound equipment available in all health facilities and providing free obstetric ultrasounds in accordance with national health policy recommendations would help ensure the systematic integration of ultrasound technology into prenatal care and improve the early detection of fetal malformations. Finally, in terms of care delivery, it is recommended that health authorities optimize the referral system to enable earlier surgical intervention during the neonatal period. This requires strengthening peripheral facilities, training healthcare personnel, and establishing a functional network for maternal and neonatal follow-up and referral. Providing targeted free healthcare for pregnant women and children under five would also improve access to specialized care, aligning with SDG 3 on health and well-being.

## Ethical approval

Ethical approval for this study was granted by the Dean’s Office of the Faculty of Health Sciences at the University of Bangui. The protocol was reviewed and validated by the department heads, unit heads, and head nurses of the Bangui Pediatric University Hospital Complex. Patient confidentiality was strictly maintained. Informed consent was obtained from parents or legal guardians for the use of medical images.

## Funding

No specific funding was received for this study. The authors declare that the preparation of this manuscript did not receive any grants, institutional support, or commercial support.

## Authors’ contributions

Bogning Mezio Olivier Brice: Manuscript drafting and overall study supervision Gbelesso Junior Sylvère: Clinical data collection and procedure monitoring

Malendele Stéphane Francia: Neonatal monitoring and infant data collection

Henko Kinding Yannick Wilson: Data analysis and postoperative follow-up

Ndoma Ngatchoukpo Valère: Surgical supervision and surgical data validation

Gody Jean Chrysostome: Supervision, study validation, and final draft oversight.

## Conflicts of interest

The authors declare that they have no conflicts of interest.

## Appendix Additional illustrations

**Figure A1:** Lumbar spina bifida with an infected meningocele, CHUPB

**Figure 2A:** Lumbar spina bifida with a non-infected meningocele, CHUPB

**Figure 3A:** Frontonasal encephalocele, CHUPB

**Figure 4A:** Occipital encephalocele, CHUPB

**Figure 5A:** Anencephaly, CHUPB

**Figure 6A:** Ruptured occipital encephalocele, CHUPB
